# D-Alanylation of Teichoic Acids and Loss of Poly-N-Acetyl Glucosamine in *Staphylococcus aureus* during Exponential Growth Phase Enhance IL-12 Production in Murine Dendritic Cells

**DOI:** 10.1371/journal.pone.0149092

**Published:** 2016-02-12

**Authors:** Lisbeth Drozd Lund, Hanne Ingmer, Hanne Frøkiær

**Affiliations:** Department of Veterinary Disease Biology, University of Copenhagen, Frederiksberg, Denmark; National Research Laboratory of Defense Proteins, REPUBLIC OF KOREA

## Abstract

*Staphylococcus aureus* is a major human pathogen that has evolved very efficient immune evading strategies leading to persistent colonization. During different stages of growth, *S*. *aureus* express various surface molecules, which may affect the immune stimulating properties, but very little is known about their role in immune stimulation and evasion. Depending on the growth phase, *S*. *aureus* may affect antigen presenting cells differently. Here, the impact of growth phases and the surface molecules lipoteichoic acid, peptidoglycan and poly-N-acetyl glucosamine on the induction of IL-12 imperative for an efficient clearance of *S*. *aureus* was studied in dendritic cells (DCs). Exponential phase (EP) *S*. *aureus* was superior to stationary phase (SP) bacteria in induction of IL-12, which required actin-mediated endocytosis and endosomal acidification. Moreover, addition of staphylococcal cell wall derived peptidoglycan to EP *S*. *aureus* stimulated cells increased bacterial uptake but abrogated IL-12 induction, while addition of lipoteichoic acid increased IL-12 production but had no effect on the bacterial uptake. Depletion of the capability to produce poly-N-acetyl glucosamine increased the IL-12 inducing activity of EP bacteria. Furthermore, the mutant *dltA* unable to produce D-alanylated teichoic acids failed to induce IL-12 but like peptidoglycan and the toll-like receptor (TLR) ligands LPS and Pam3CSK4 the mutant stimulated increased macropinocytosis. In conclusion, the IL-12 response by DCs against *S*. *aureus* is highly growth phase dependent, relies on cell wall D-alanylation, endocytosis and subsequent endosomal degradation, and is abrogated by receptor induced macropinocytosis.

## Introduction

The Gram-positive bacterium *Staphylococcus aureus* often colonizes the human skin and mucosal surfaces without causing any symptoms. However, if the epithelial layer is compromised, there is a significant risk of serious infection, since *S*. *aureu*s often evades elimination by the immune system [1]. This may be due to the fact that many staphylococcal strains have evolved mechanisms to circumvent innate immune responses, by expressing cell wall-anchored proteins, extracellular polysaccharides, and toxins such as hemolysins [2]. The growth conditions are highly important for the timing of expression and secretion of such virulence factors [3]. In exponential growth phase (EP) surface-attached adhesion molecules are expressed and facilitate colony establishment. In addition, teichoic acids anchored within a thick peptidoglycan meshwork, are D-alanine-esterified during exponential phase mediated by four proteins encoded by the *dltABCD* operon, thus contributing to a net positive charge of the cell wall, which mediates binding to implants or host surfaces [4–6]. During post-exponential phase, some *S*. *aureu*s strains are capable of forming polysaccharide capsules that have been shown to mediate adhesion to endothelial cells and obstruct endocytosis [7–9]. Also, many strains secrete extracellular polysaccharides, such as poly-N-acetyl glucosamine (PNAG), which is encoded by the intercellular adhesin (*ica*) operon and facilitates aggregation of late exponential phase bacteria involved in biofilm formation [10, 11]. All these factors are believed to contribute to the immune evasion; however, specific mechanisms involved are only poorly understood.

Dendritic cells (DCs) are sentinel cells residing in peripheral tissues, where they play an important role in orchestrating the innate and adaptive immune responses [12, 13]. DCs have recently been shown to play key role in *S*. *aureus* clearance by producing IL-12 [14]. IL-12 production in concert with T cells or natural killer (NK) cells leads to production of interferon (IFN)-γ, a cytokine essential for efficient endocytosis by macrophages and subsequent reduction of bacterial load [15, 16]. Accordingly, the IL-12 induction in DCs represents a key element in an efficient combat of staphylococcal infections. We and others have previously shown that some Gram-positive as well as Gram-negative bacteria are capable of inducing IFN-β upon endocytosis in DCs, and IFN-β in turn induces IL-12 [17–19]. IL-12 may however, also be induced directly without the involvement of IFN-β [19]. DCs and other immune cells express a palette of pattern recognition receptors (PRRs) that facilitates binding of specific microbial structures, such as carbohydrates, lipoproteins and nucleic acids, all bacterial components important for pathogen survival [20]. The most studied PRRs comprise the toll-like receptors (TLRs). TLR2 deficient mice are more susceptible to infection than wild type mice, signifying the involvement of TLR2 in initiation of a protective immune response towards *S*. *aureus* [21]. Several studies have shown that the cell wall constituents, lipoteichoic acid (LTA), wall teichoic acid (WTA), lipoprotein, and peptidoglycan (PGN) are ligands for TLR2 [22–24]. Newer studies using knock out strains and more thorough purification, however, have provided strong evidence against WTA, LTA and PGN as TLR2 ligands by demonstrating that it is the lipoprotein present in these preparations that binds to TLR2 [25, 26]. Stimulation with the cell wall fragments leads to the induction of TNF-α and IL-6, but not IL-12 [22, 23]. The D-alanylated form of WTA and LTA is prevailing in EP *S*. *aureus* and induces TNF-α in human whole blood more efficiently than LTA without the amino acid [5, 27]. The scavenger receptor CD36 was shown to bind LTA in turn promoting internalization of *S*. *aureus* without directly inducing cytokine production [28]. Hence, different mechanisms may be involved in uptake of and response to *S*. *aureus*, but how and whether the different mechanisms induce different cytokine responses has not been unraveled.

IL-12 is known to be indispensable for an efficient clearance of *S*. *aureus*, but IL-12 production in DCs upon *S*. *aureus* stimulation has not been thoroughly addressed. As TLR2 seems to be important for *S*.*aureus* stimulated induction of IL-12 and since the accessibility of TLR2 stimulating ligands may be of major importance for IL-12 production, we hypothesized that LTA/WTA and in particular D-alanylated LTA/WTA and carbohydrate structures such as PNAG, all molecules exposed on *S*. *aureus*, may play key roles in the induction of IL-12, either through stimulation of PRRs, or by affecting the uptake of bacteria. Moreover, various carbohydrate structures may play a role in the microbe-host cell interaction, e.g. by concealing the bacteria. Exposure of these molecules depends to a large extend on the growth phase of the bacteria. We therefore hypothesized that the growth phase-dependent expression of some of these surface molecules on *S*. *aureus* would influence the induction of IL-12 and IFN-β in DCs, and thus determine the immunological response.

In the present study, we found that exponential phase *S*. *aureus* induced a strong IL-12 response while stationary phase bacteria did not induce IL-12 and only a weak or moderate induction of other cytokines. The induced IL-12 response depended on the uptake and acidification of intact bacteria and involved D-alanylated teichoic acid dependent endocytosis, while exposure of PGN dampened the IL-12 response by increasing macropinocytosis.

## Materials and Methods

### Bacterial strains

The following strains were used in this study: *Staphylococcus aureus* (8325–4 [29], 8325–4 Δ*ica*:*tet* [1]), 15981[30], 15981 Δ*ica*:*tet* [31], SA113 [32], SA113*Δdlt* [33], and RN6911[34]), *Lactobacillus acidophilus* NCFM (Danisco, Copenhagen, Denmark), and *Escherichia coli* Nissle 1917 O6:K5:H1 (Statens Serum Institut, Copenhagen, Denmark). *S*. *aureus* strains were grown in 20 ml tryptic soy broth (TSB) in an Erlenmeyer flask (100 ml flasks) or agar containing 10% sheep blood overnight (ON) at 37°C with agitation (130 rpm). Next, the ON culture was diluted 1:20 in fresh warm TSB and incubated at 37°C, shaking 130 rpm until exponential phase was reached (*A*_*600*_ 0.8, 2–4 h incubation). Stationary and exponential phase bacteria were washed twice in sterile PBS and plated on TSA for colony forming unit counting. To UV-inactivate *S*. *aureus*, exponential and stationary bacteria were adjusted to an *A*_*600*_ of 0.5 in 20 ml PBS and subjected to UV radiation (monochromatic wavelength of 254 nm; CL-1000 cross-linker; UVP, Cambridge, United Kingdom) by pulsed UV radiation of 6 sec per pulse for a total of 90 sec. Viability of the bacteria was confirmed by plating the UV-irradiated bacteria on TSA plates. To obtain lysed bacteria, UV-irradiated bacteria were treated with 50 μg/ml lysostaphin (Sigma-Aldrich, L7386) for 15 min at 37°C, and kept at -80°C until use. Bacterial culture supernatant was harvested from exponential and stationary phase and filtered through 0.2 μm pore-size filters. For endocytosis experiments *S*. *aureus* was fluorescently labeled using AlexaFluor647 conjugated succinimidyl-ester (Molecular Probes, Eugene, OR, A-20006) *L*. *acidophilus* and *E*. *coli* were grown as previously described [19], with the exception of UV-treatment.

### Generation of bone marrow derived dendritic cells

Bone marrow were collected from C57BL/6 mice (Taconic, Lille Skensved, Denmark) by flushing the tibia and femur with ice cold DPBS and washed twice in DPBS [35]. Subsequently, 3x10^5^ bone marrow cells were seeded into Petri dishes in 10 ml of RPMI 1640 (Sigma-Aldrich St. Louis, MO) containing 10% (v/v) heat-inactivated fetal calf serum (Cambrex Bio Whittaker, Charles City, IA), supplemented with glutamine (4mM) penicillin (100 U/ml; Lonza), streptomycin (100 μg/ml; Lonza), 50 μm 2-mercaptoethanol (Gibco) and 15 ng/ml GM-CSF (harvested from a GM-CSF-transfected Ag8.653 myeloma cell line) The cells were refreshed with complete medium containing 15 ng/ml GM-CSF on day three and six, and ready to use on day eight. CD11c+ DCs were analyzed by flow cytometry with a purity of approximately 80%.

All animals used as source of bone marrow cells were housed under conditions approved by the Danish Animal Experiments Inspectorate (Forsøgdyrstilsynet) according to The Danish Animal Experimention Act; LBK no. 474 from 15/05/2014, and experiments were carried out in accordance with the guidelines of ‘The Council of Europe Convention European Treaty Series (ETS)123 on the Protection of Vertebrate Animals used for Experimental and other Scientific Purposes’.

### Ligands and inhibitors

The following ligands were used at the final concentrations indicated: staphylococcal PGN and LTA 1 and 10 μg/ml (Sigma Aldrich, St. Louis, MO), LPS 1 μg/ml (Sigma Aldrich, St. Louis, MO), Pam3CSK4 1 μg/ml, Pam2CSK4 1 μg/ml (Cayla, Invivogen, Tolouse, France). The inhibitor Cytochalasin D (Sigma Aldrich, C2618) was used at a final concentration of 0.5 μg/ml, Bafilomycin A1 (Invivogen) 50nM, and Chloroquine (Invivogen, tlrl-chq) 10 μM.

### Stimulation of dendritic cells with bacteria and ligands

Immature DCs were harvested and adjusted in complete medium to a concentration of 2x10^6^ cells/ml. Cells were seeded 1x10^6^ into 48-well plates (NUNC, Roskilde, Denmark) and treated with strains of *S*. *aureus* at the indicated multiplicity of infection (MOI), *L*. *acidophilus* (10 μg/ml) or *E*. *coli* (10 μg/ml). For pre-stimulation experiments, *S*. *aureus* were added to the cells 30 min prior the first stimuli. The cells were incubated at 37°C and 5% CO_2_. Cytochalasin D was added to the cells 1 h before stimulation with ligands or bacteria.

### Cytokine production by dendritic cells

DCs were incubated for 20 h and cell culture supernatant was harvested. Levels of IL-12p70 (DY419), TNF-α (DY410) and IL-10 (DY417) (all purchased from R&D Systems, Minneapolis, MN, USA) were detected by commercially available enzyme linked immunosorbent assay (ELISA) kits according to the manufacturer’s instructions.

### RNA extraction, cDNA synthesis and RT-PCR analysis

DCs were harvested at various stimulation time-points and total RNA was extracted using the MagMAX-96 Total RNA Isolation Kit (AM1830, Applied Biosystems) on a MagMAX Express Magnetic Particle Processor (Applied Biosystems). RNA concentration and purity were measured on a Nanodrop (Thermo, Wilmington, USA). cDNA was produced from 500 ng total RNA by the use of High-Capacity cDNA Reverse Transcriptase Kit (Applied Biosystems) according the manufacturers’ instructions. The expression of the genes encoding IFN-β, IL-10 and β-actin was detected using primers and probes as previously described [36]. The amplification was performed on 2 μl cDNA (3 ng/μl) in duplicates on the StepOnePlus instrument by using fast thermal cycling parameters and 1xTaqMan Universal PCR Master Mix (all from Applied Biosystems) in a total reaction volume of 10 μl. Relative transcript levels were calculated by the comparative cycle threshold (C_T_) method [37]. Fold change of gene expression was calculated by the ΔΔC_T_ method where the expression of target genes was normalized to β-actin as the reference gene, and fold change was calculated relative to the average ΔC_T_ from control samples at all time-points.

### Endocytosis assay

Immature DCs (2x10^6^) were pre-treated with Cytochalasin D (0.5 μg/ml) for 1 h at 37°C, and seeded (3x10^5^) in 96-well U-bottom tissue culture plates (NUNC, Roskilde, Denmark). Next, the cells were stimulated with *S*. *aureus* or ligands for 30–60 min and subsequently chased with 500 μg/ml fluorescein isothiocyanate (FITC)-conjugated dextran (150 kDa, Sigma-Aldrich) for 10 min at 37°C. For pre-stimulation, cells were pre-treated with ligands for 30 min followed by fluorescently labeled *S*. *aureus* stimulation for 30 min and then chased with FITC-dextran for 10 min. The cells were washed twice in ice-cold DPBS containing 1% (v/v) FCS and 0.15% (w/v) sodium azide (PBSAz) and fixed in PBSAz supplemented with 1% (v/v) formalin. Uptake of bacteria and FITC-dextran was analyzed by BD FACSCanto II flow cytometer (BD Biosciences, San Jose, CA) based on counting 10,000 cells. The levels of uptake were expressed as the geometric mean fluorescence intensity (MFI) and percent of events.

### Maturation of DCs and Cell Viability

For maturation, DCs (2x10^6^ cells/ml) were stimulated as for ELISA and supernatants were discarded. Next, cells were washed twice in PBSAz, incubated with anti-mouse CD16/CD32 BD Fc Block (3 μg/ml; BD Biosciences, San Jose, CA, 553141) for 10 min on ice to block unspecific binding. Subsequently the cells were stained with monoclonal anti-mouse CD40-PE (2μg/ml, 12–0401), CD86(B7-2)-APC (1.25 μg/ml,17–0862) (both from eBiosciences) and CD80(B7-1)-FITC (1.25 μg/ml; BD Pharmingen, San Diego, CA, 553768) for 45 min at RT. Cells were then washed twice in PBSAz, fixed and analyzed by flow cytometry. MFI was used as a measure of maturation based on counting 10,000 cells. To analyze cell viability, the DCs were washed twice in ice-cold DPBS after 1 or 20 h of stimulation with bacteria and their supernatant. Next, the cells were re-suspended in BD Binding buffer, stained with anti-mouse Annexin V-PE and 7-AAD-PerCpCy5.5 (BD Pharmingen, San Diego, CA, 559925) for 15 min at RT and analyzed by flow cytometry based on counting 5,000 cells. Percentages of apoptotic, dead or double positive cells were used. Data analysis was performed using FlowJo Version 10 software (Treestar Inc).

### Statistics

GraphPad Prism 5.0 software (San Diego, CA) was used to perform one-way ANOVA with Bonferoni as post-test, or Student's t test. P-values of <0.05 were considered significant.

## Results

### *S*. *aureus* in exponential, but not in stationary phase induces high cytokine production in DCs

**Fig 1:**
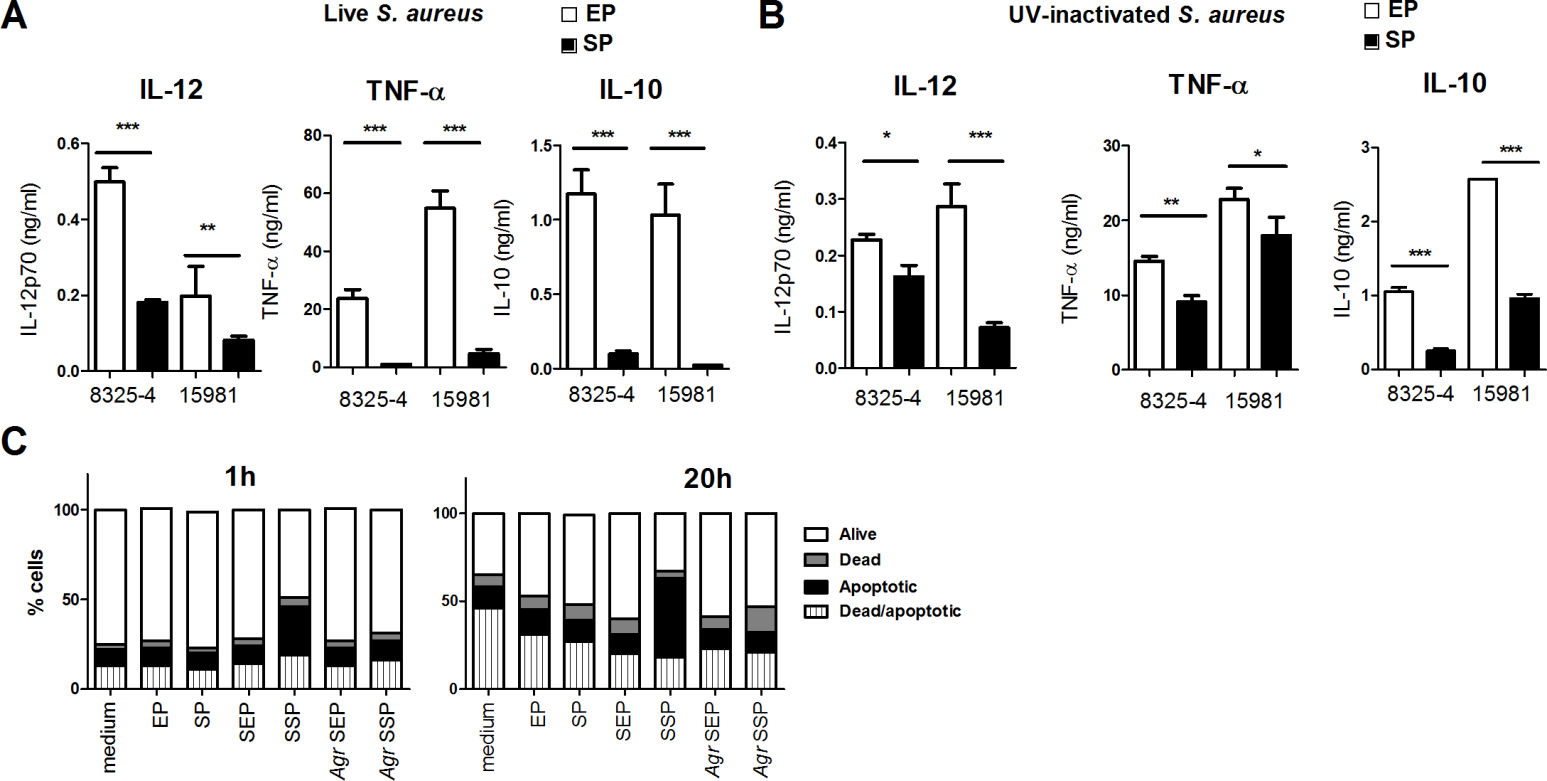
Exponentially growing S. aureus strains are strong IL-12 inducers. (A) DCs were stimulated with live *S*. *aureus* 15981 or 8325–4 in exponential or stationary phase with a MOI 10. Production of IL-12p70, TNF-α and IL-10 was detected in the supernatants by ELISA after 20h of stimulation. Data shown represents one representative experiment of three. Means and SD are based on technical replicates. (B) DCs were stimulated for 20h with UV-inactivated 8325–4 and 15981 in exponential or stationary phase with a MOI of 6. At least three experiments were performed. C) DC viability was analyzed by flow cytometry after 1h or 20h of stimulation with UV-inactivated strain 8325 in exponential (EP) or stationary (SP) phase, or 8% supernatant harvested from 8325–4 or RN6911 in EP (SEP) or SP (SSP). The amount of alive, apoptotic, dead or cells double positive for dead and apoptotic cells are shown as percentages based on counting 5000 cells.

The expression of molecules associated with survival and virulence of *S*. *aureus* is highly growth phase-dependent. Here, we set out to compare the immune stimulatory effects of the laboratory strain 8325–4 and the clinical strain 15981 harvested in either exponential (EP) or stationary (SP) growth phase, respectively, on murine bone marrow-derived DCs (Fig 1). First, we determined the cytokine response against live *S*. *aureus* by measuring the levels of IL-12, TNF-α and IL-10 in supernatants harvested from DCs stimulated 20 h earlier. DCs reacted in a dose-dependent manner; the cytokine production increased with an increasing MOI up to a maximum level and then dropped again (data not shown). The dose-response curves varied between strains, mice and cytokines tested. A MOI of 5 was chosen because it induced detectable cytokine levels for all the strains tested. EP bacteria strongly up-regulated the production of IL-12, TNF-α and IL-10 as compared to the SP bacteria, which generally were poor cytokine inducers (Fig 1A). To avoid bacterial growth and production of toxins during experiments, we stimulated with UV-irradiated 8325–4 and 15981 in EP or SP. The UV-treatment of the bacteria hardly affected their cytokine induction capacity, and the difference in cytokine profile induced by EP and SP bacteria was maintained (Fig 1B). Many cytolytic molecules, such as phenol soluble modulins and hemolysin-α, are produced during SP under the control of the accessory gene regulator (*agr*) gene promoter, and we thus speculated whether the observed decrease in cytokines in SP could be attributed to DC cell death induced by *S*. *aureus* produced toxins. Thus, we investigated the lytic capability of intact *S*. *aureus* and spent medium harvested from the bacteria on DCs (Fig 1C). Strain 8325–4 in EP and SP did not increase the amount of dead DCs after 1h or 20h of stimulation, nor did the spent medium from EP bacteria compared to DCs stimulated with medium. Only supernatant harvested from 8325–4 in SP led to an increase in the amount of apoptotic and dead cells, indicating that the production of cytotoxic effector molecules, when secreted induces cell death. Most importantly, however, this demonstrated that we could rule out cell death and a role of the toxins, as the cause of the weak (almost absent) induction of cytokines by SP bacteria.

### Uptake and endosomal acidification is required for *S*. *aureus*-induced IL-12

**Fig 2:**
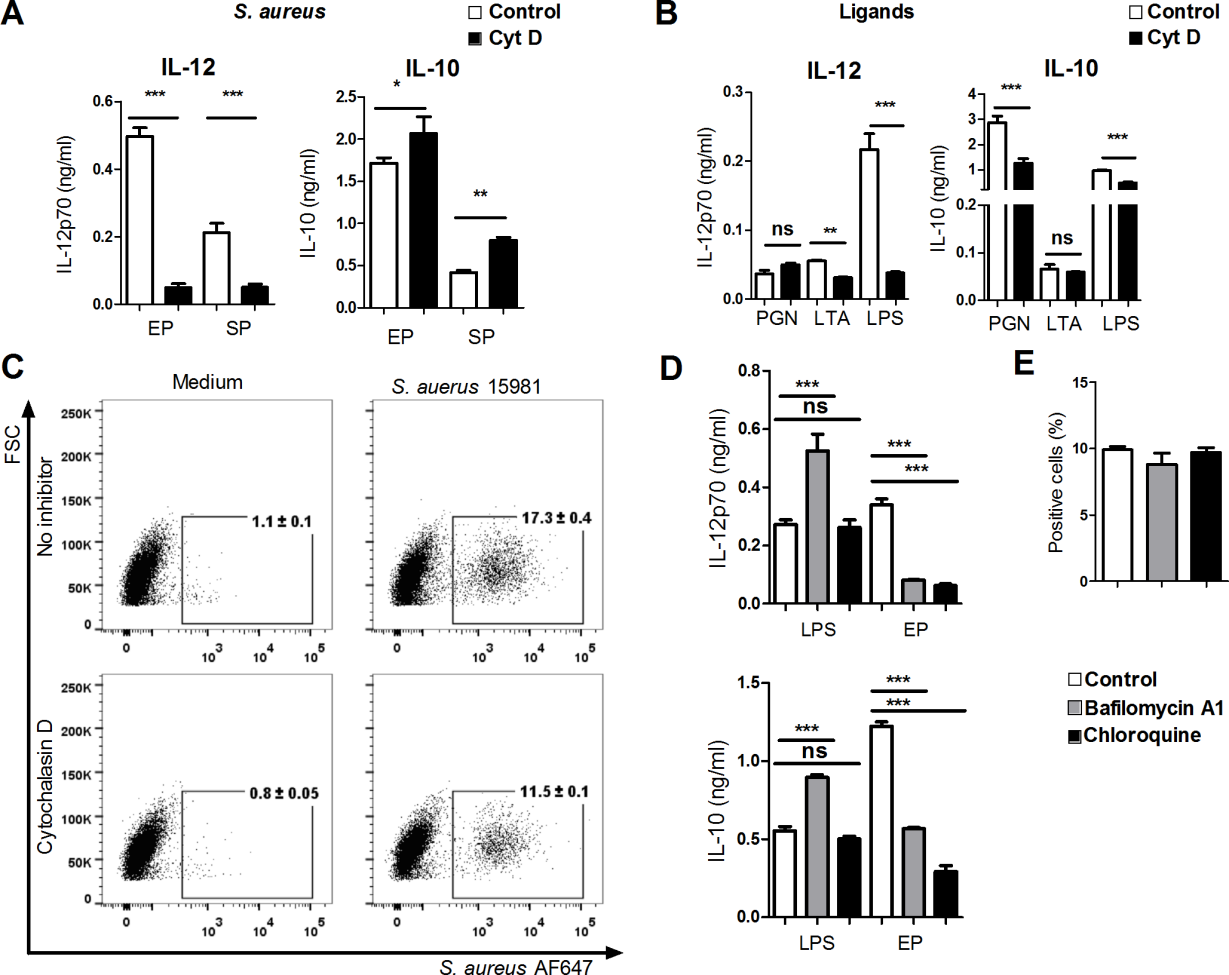
Phagocytosis and acidification is important for *S*. *aureus* induced IL-12 production. (A) DCs were pre-treated for 1h with 0.5 μg/ml cytochalasin D before stimulation with UV-inactivated 8325–4 in exponential or stationary phase. ELISA was used to detect the levels of IL-12p70 and IL-10 in the supernatant after 20h. Means and SD are based on technical replicates. Data represent one out of three experiments. (B) DCs were incubated with cytochalasin D (0.5 μg/ml) for 1h and subsequently stimulated wit PGN (10 μg/ml), LTA (10 μg/ml) or LPS (1 μg/ml). IL-12p70 and IL-10 were measured in the supernatant after 20h. Means and SD are based on technical replicates. Data represents one out of three experiments. (C) DCs pre-incubated with 0.5 μg/ml cytochalasin D were subsequently stimulated for 1h with AlexaFluor647 labeled *S*. *aureus* 15981 in exponential phase (MOI 2). The uptake was measured by flow cytometry. Numbers indicate the percentages of AlexaFluor647 positive cells. (D) Bafilomycin A1 (50nM) or chloroquine (10 μM) were added to the DCs for 1h. Subsequently, DCs were stimulated with LPS (1 μg/ml) or *S*. *aureus* 15981 in EP. After 20h IL-12p70 and IL-10 were measured in the supernatant. (E) DCs were pre-treated for 1h with bafilomycin A1 (50 nM) or chloroquine (10 μM) followed by 30 min of stimulation with AlexaFlour647 labeled *S*. *aureus* 15981 in EP (MOI 2). AlexaFlour647 positive cells were measured by flow cytometry. Data are based on 10,000 cell counts and double cells were excluded by gating FSC-A/FSC-H. (error bars, SD; ns, no significant difference; * p<0.05, ** p< 0.01 and ***p<0.001).

Endocytosis of *S*. *aureus* by macrophages is a prerequisite for TNF-α and IL-6 production [38], but the dependency of endocytosis in DCs for induction of IL-12 has not been examined, nor has the role of the endocytosis for the cytokine profile been elucidated. To determine the role of staphylococcal uptake by DCs for induction of IL-12, IL-10, IL-6 and TNF-α, we inhibited actin-dependent endocytosis with Cytochalasin D (Fig 2A). IL-12 production in response to EP *S*. *aureus* was almost completely abrogated after inhibition of actin-mediated uptake, while IL-10 production was slightly enhanced. TNF-α production was partially dependent on uptake of EP bacteria, but not on uptake of SP bacteria (data not shown). Furthermore, inhibiting actin dependent uptake reduced the IL-12 production induced by LPS and LTA. PGN did not induce IL-12, but induced as LPS a high level of IL-10, which was to some degree affected by Cytochalasin D (Fig 2B). To assess the capability of DCs to take up *S*. *aureus*, we added AlexaFluor-labeled EP bacteria to the DCs (Fig 2C). By comparing the uptake of bacteria in DCs with or without pre-treatment with Cytochalasin D, we could exclude cells with bacteria adhered to the surface and found that about 6% of the DCs had endocytosed bacteria. To determine whether acidification of the endosome required for degradation of *S*. *aureus* was indispensable for the following IL-12 production, we perturbed endosome acidification with chloroquine or bafilomycin 1. Chloroquine blocked EP *S*. *aureus* induced–but not LPS induced IL-12 and IL-10 production (Fig 2D). Bafilomycin 1 also blocked *S*. *aureus* induced cytokine production, but led to increased production upon LPS stimulation. Blocking acidification did not influence the uptake of *S*. *aureus* (Fig 2E). Together these data indicate that endocytosis of *S*. *aureus* as well as endosomal acidification to reveal PRR ligands within the endosome is needed to initiate an IL-12 response.

### *S*. *aureus* induces *ifn-β* expression and does not induce increased macropinocytosis

**Fig 3:**
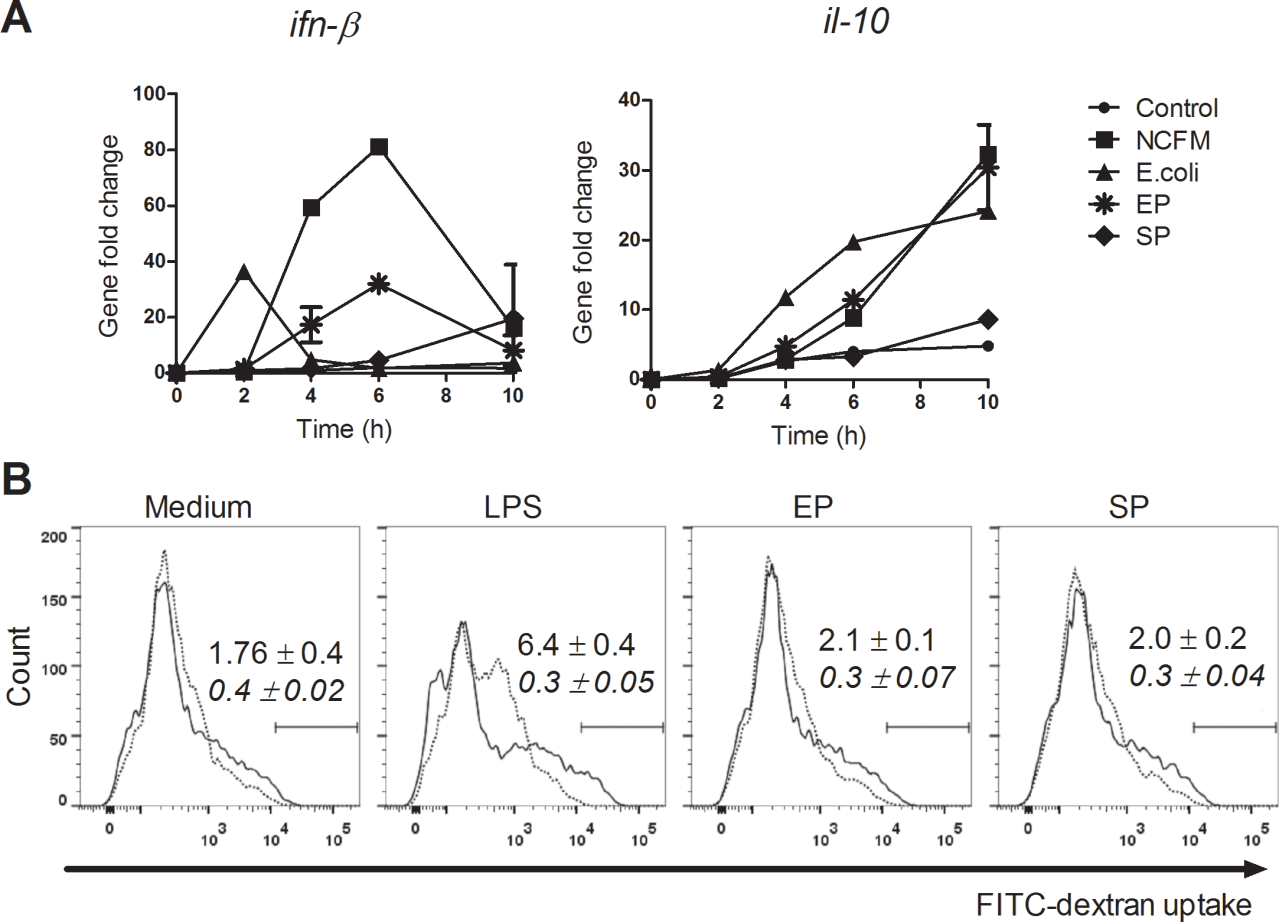
*S*. *aureus* induces *inf-β* expression but does not induce increased macropinocytosis. (A) DCs were stimulated with UV-killed 8325–5 in exponential or stationary phase (MOI 6), *L*. *acidophilus* NCFM (10 μg/ml) or *E*. coli Nissle (10 μg/ml). Expression of i*fn-β* and i*l-10* was analyzed by qPCR and normalized to β-actin after 2, 4, 6 and 10h. Means and SD are based on technical replicates. Data represent one out of three experiments. (B) DCs were pre-treated with or without cytochalasin D (0.5 μg/ml) for 1h. Next, cells were added complete DC medium, LPS (1 μg/ml) or 15981 in exponential or stationary phase (MOI 2) for 1h together with FITC-coupled dextran particles (500 μg/ml). Uptake of dextran particles was measured by flow cytometry. Data are based on the count of 10,000 cells and double cells were excluded by FSC-A/FSC-H. Numbers indicate mean and standard deviations of the geometrical mean fluorescence intensity of three technical replicates (upper numbers represents the solid line of cells with no inhibitor, and the lower numbers represent dotted line of cells incubated with cytochalasin D).

We have previously shown that the Gram-positive *Lactobacillus acidophilus* NCFM and the Gram-negative *Escherichia coli* Nissle 1917 induce IFN-β and IL-10 production in DCs with distinct kinetic profiles [19]. Also, IFN-β production in turn leads to a potent production of IL-12 [19]. To establish whether the Gram-positive *S*. *aureus* follows the same kinetic as the potent Gram-positive IL-12 inducer *L*. *acidophilus* NCFM, we stimulated DCs with *L*. *acidophilus* NCFM, *E*. *coli* Nissle 1917 as well as EP and SP *S*. *aureus* 8325–4 (Fig 3A). Indeed, *S*. *aureus* in EP induced the slow expression of *ifn-β* and *il-10* as *L*. *acidophilus* NCFM but different from *E*. *coli* Nissle 1917, although the *ifn-β* expression was lower. The SP *S*. *aureus* induced almost no expression of *ifn-β* and only a small change in *il-10* expression. Compared to the Gram-positive bacteria, *E*. *coli* induced faster *ifn-β* and *il-10* expression. These data show that the cytokine responses against the Gram-positive bacteria are initiated with a delay and may reflect when and where the involved PRRs are triggered to induce cytokine expression. Addition of TLR ligands or bacteria exposing TLR ligands induces increased macropinocytosis in DCs [39] and, to assess whether the *S*. *aureus* induces macropinocytosis, we stimulated DCs with *S*. *aureus* in EP, SP or with LPS, and subsequently added FITC-dextran (Fig 3B). Immature DCs constitutively take up dextran particles by macropinocytosis, indicated by the populations with a high-intensity FITC that is lost after addition of Cytochalasin D. Neither EP nor SP *S*. *aureus* increased the uptake of FITC-dextran, which is comparable to results obtained after *L*. *acidophilus* NCFM stimulation (data not shown) and indicative of the absence of TLR-ligands on the surface of *S*. *aureus*. In contrast, LPS that is known as a potent inducer of macropinocytosis [39] increased the uptake of FITC-dextran (Fig 3B). Taken together, these data indicate that, as EP and SP *S*. *aureus* do not induce increased macropinocytosis, they do not expose TLR ligands on their surface.

### Intact *S*. *aureus* is required for IL-12 production and full maturation of DCs

**Fig 4:**
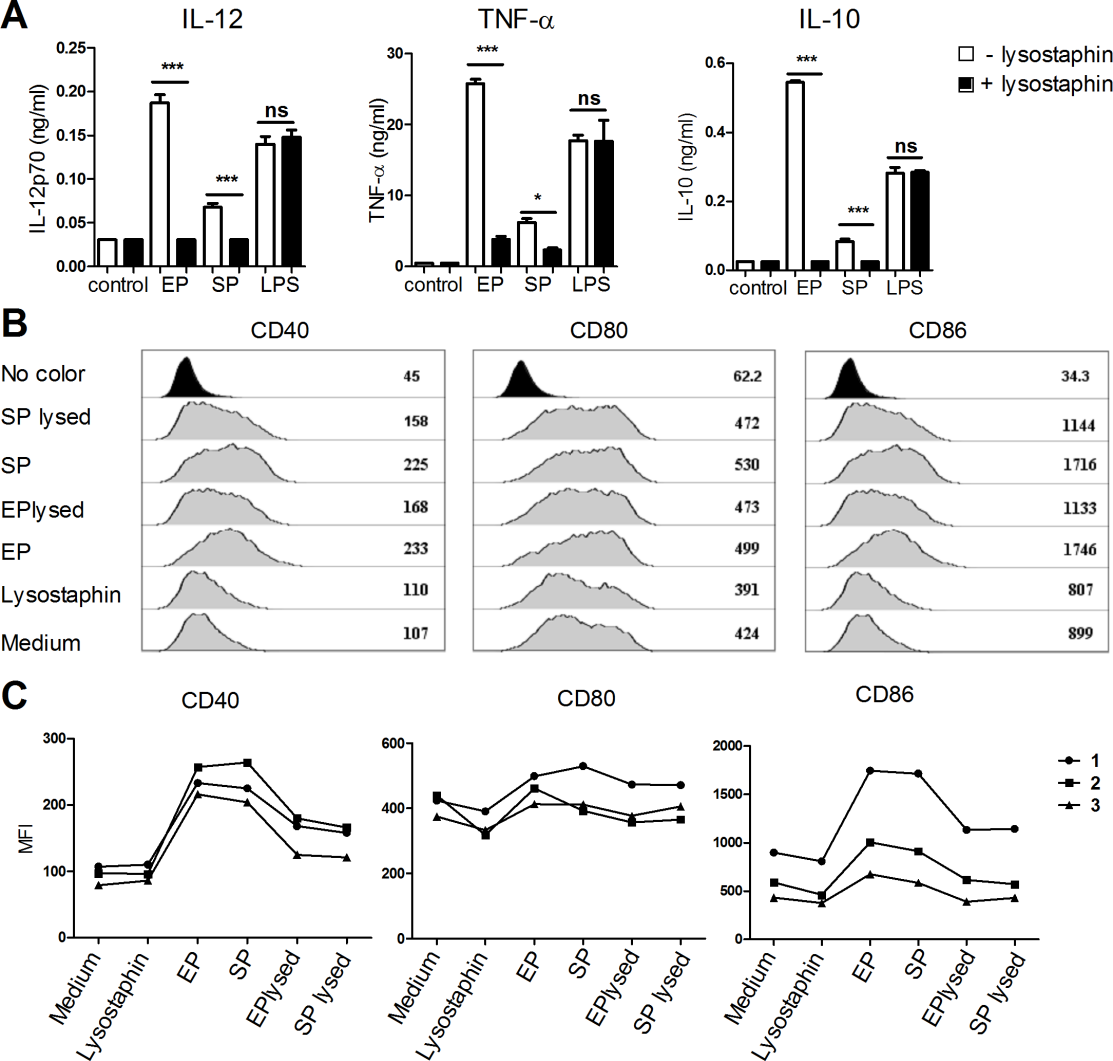
Only intact *S*. *aureus* is able to fully mature dendritic cells. (A) Intact or lysostaphin lysed UV-inactivated 8325–4 (MOI 6) or LPS (1μg/ml) +/- lysostaphin were subjected to DCs. After 20h of stimulation, IL-12p70, TNF-α and IL-10 levels were detected in the supernatant by ELISA. (B) Mean fluorescence intensity (MFI) of CD40, CD80 and CD86 were detected by flow cytometry after maturation of DCs for 20h with intact or lysed 8325–4 in exponential or stationary phase. Means and SD are based on technical replicates. Data represent one experiment out of three. (C) As in B, combination of three experiments. (error bars, SD).

We have previously shown that for the lactobacilli, intact bacteria are required for full maturation of DCs [23]. Accordingly, we aimed to investigate if this is also the case for *S*. *aureus* induced maturation. UV-inactivated *S*. *aureus* (strain 8325–4) harvested in EP and SP was lysed by lysostaphin, an antibacterial endopeptidase that cleaves pentaglycin cross bridges of the major cell wall component PGN [40], potentially resulting in the exposure of the TLR2 binding lipopeptide. The capacity of lysed *S*. *aureus* to induce cytokines in DCs was subsequently compared to intact bacteria. Only intact *S*. *aureus* induced IL-12 and IL-10 production, while fragmented bacteria induced a minor TNF-α production compared to intact bacteria (Fig 4A). Stimulation with LPS was not affected by lysostaphin, thus demonstrating that lysostaphin does not affect the cytokine production in DCs. To further investigate the maturation of DCs, the expression of co-stimulatory molecules CD40, CD80 and CD86 was analyzed by flow cytometry (Fig 4B). Compared to intact *S*. *aureus*, fragmented *S*. *aureus* were as efficient at inducing CD80 expression, whereas CD40 and CD86 expression was much higher in DCs exposed to intact bacteria, independent of the growth phase (Fig 4B and 4C). Thus, independent of growth phase intact *S*. *aureus* bacteria provide potent maturation of DCs but only EP *S*. *aureus* induces appreciable amounts of IL-12.

### PGN, but not LTA induces increased macropinocytosis and abrogates IL-12 production

**Fig 5:**
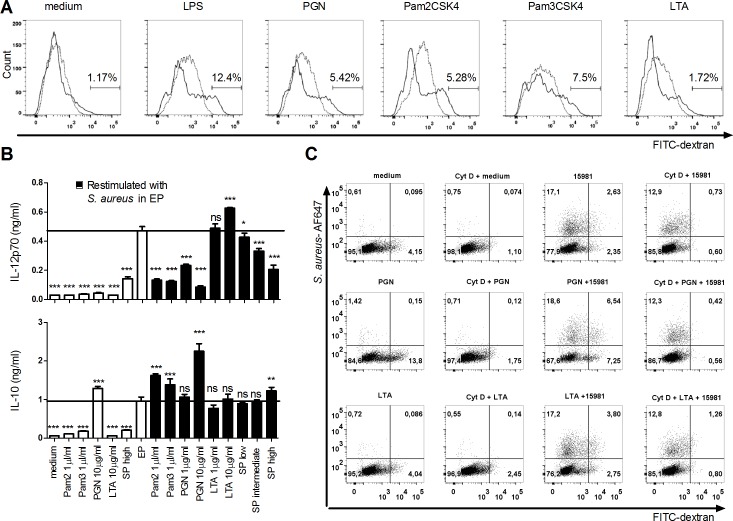
Staphylococcal PGN, but not LTA induce macropinocytosis and abrogate IL-12 production. (A) DCs was stimulated with, LPS, Pam2CSK4, or Pam3CSK4 (1 μg/ml) or PGN or LTA (10 μg/ml) for 30 min with or without pre-treatment with cytochalasin D. Then, cells were chased with FITC-coupled dextran (500 μg/ml) for 10 min. Dextran uptake was measured by flow cytometry by counting 10,000 cells. Numbers indicate the percentages of FITC-positive cells. (B) DCs were pre-stimulated with PGN or LTA (1 and 10 μg/ml), LPS, Pam2CSK4, Pam3CSK4 (1 μg/ml), or *S*. *aureus* SA113 in stationary phase (MOI for: High 3; Intermediate, 2; Low, 1) for 30 min. Next, the DCs were subjected to *S*. *aureus* SA113 in exponential phase (MOI 6), (Black bars), or DC’s were stimulated with the ligands alone (white bars). After 20h, IL-12 and IL-10 levels were measured in the supernatant by ELISA. (C) DCs was pre-treated with cytochalasin D for 1h and subsequently stimulated with DC complete medium, PGN or LTA (10 μg/ml) for 30 min. Next, the cells were stimulated with AlexaFluor647 labeled *S*. *aureus* 15981 in exponential phase for 30 min, and chased with FITC-dextran for 10 min. Dot plots are based on 10,000 cells counted on FACS CantoII and single cell gating by the use of FSC-A/FSC-H. Means and SD are based on technical replicates. Data represent one out of three experiments.

West et al. demonstrated that single TLR ligands such as Pam3CSK4 and LPS induce increased macropinocytosis [39] and we have seen that these ligands concomitantly inhibit IL-12 and IFN-β induction by some intact Gram-positive bacteria (unpublished data). Both LTA and PGN preparations have been reported to stimulate TLR2 due to the content of lipoprotein [41–45]. We therefore assessed the capacity of PGN and LTA preparations from *S*.*aureus* to induce macropinocytosis and inhibit *S*. *aureus* induced IL-12. PGN induced increased macropinocytosis to a level comparable to the induction by the TLR2 ligands Pam3CSK4 and Pam2CSK4, but lower than the level induced by the TLR4 ligand LPS (Fig 5A). In contrast, LTA was unable to induce increased macropinocytosis. Pretreatment with the ligands prior to stimulation with the intact EP *S*. *aureus* demonstrated that PGN to the same degree as the PamCSK molecules inhibited the IL-12 production, but conversely enhanced IL-10 production (Fig 5B). In contrast, LTA weakly increased the IL-12 production, while showing no effect on the IL-10 production. Although neither EP nor SP bacteria increased macropinocytosis (Fig 3B), *S*. *aureus* harvested in SP was also able to inhibit the EP *S*. *aureus*-induced IL-12 induction in a dose dependent manner. In order to assess the uptake of bacteria in cells pretreated with LTA, PGN and Pam3CSK4, we pre-stimulated DCs with these ligands, then added fluorescence-labeled EP bacteria and FITC-dextran and measured the uptake by flow cytometry (Fig 5C). As expected, PGN but not LTA increased the uptake of FITC-dextran and, in the presence of bacteria, the number of cells that had endocytosed bacteria doubled (from 6% to around 12% in the PGN treated cells). Of note, half of the PGN treated cells that had endocytosed bacteria also had a high content of FITC-dextran, as indicated by the high florescence intensity. This may indicate that cells have taken up the bacteria through macropinocytosis, as opposed to phagocytosis. Pretreatment with LTA did not affect dextran or bacterial uptake. Taken together, these results demonstrated that in contrast to LTA, PGN is capable of abrogating IL-12 production and induces macropinocytosis in line with the Pam2CSK4 and Pam3CSK4.

### Extracellular polysaccharides conceal key molecules for IL-12 induction

**Fig 6:**
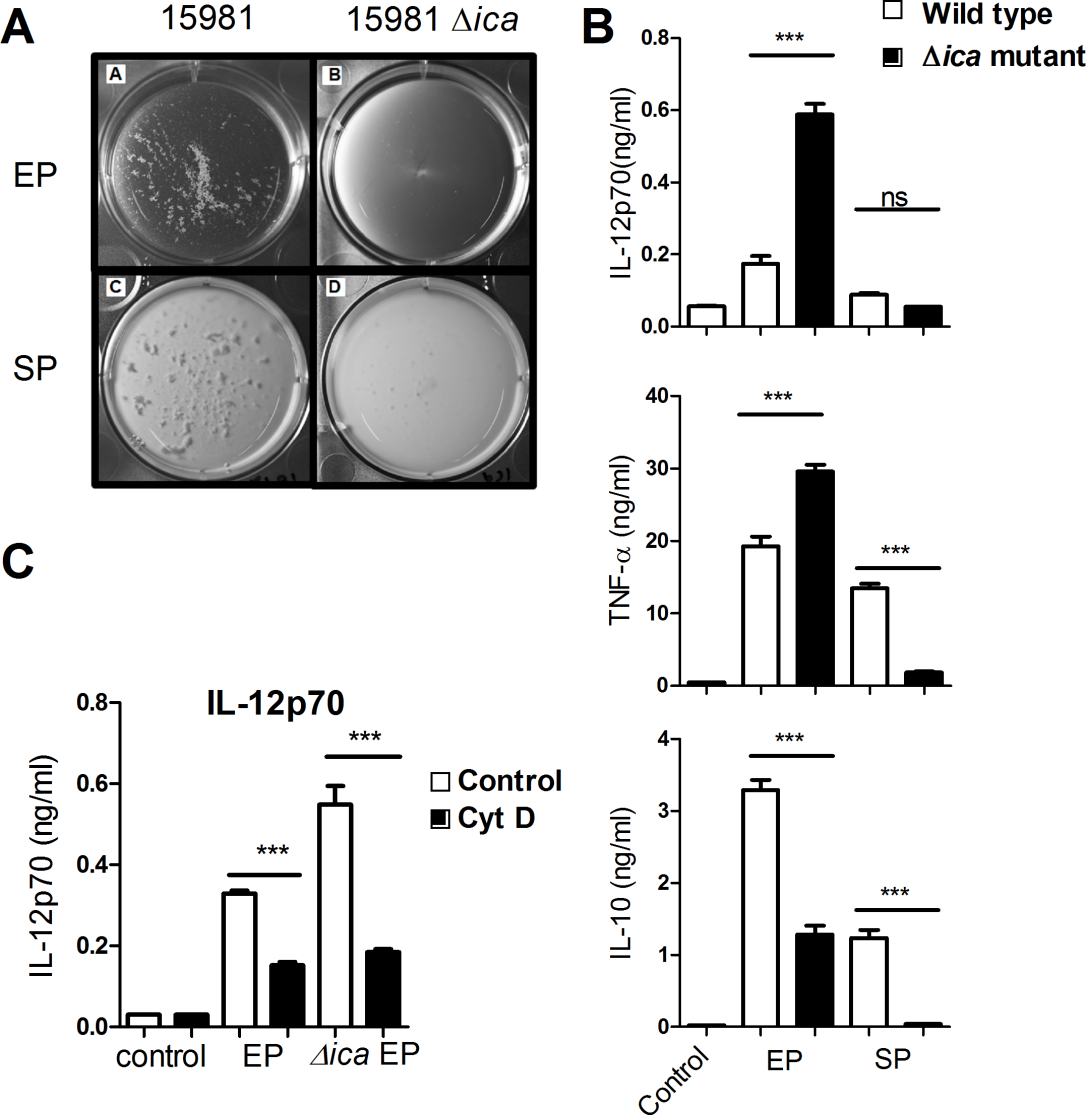
Extracellular polysaccharides impair pro-inflammatory response. (A) *S*. *aureus* strain 15981 was grown in TSB ON at 37°C, 130 rpm. The overnight culture was diluted 1/20 in fresh TSB and grown for 2h 30 min. (B) DCs were stimulated with UV-inactivated strain 15981 or its isogenic *ica* mutant in exponential or stationary phase. After 20h IL-12p70, TNF-α and IL-10 were detected in the supernatant by ELISA. Data represents one experiment out of three. (C) Phagocytosis was inhibited by treatment with cytochalasin D (0.5 μg/ml) for 1h and subsequently DCs were stimulated and analyzed as in B. Means and SD are based on technical replicates. Data show one out of three experiments. (error bars, SD; ns, no significant difference; *, ** and ***p<0.05).

Extracellular polysaccharides, such as PNAG are produced in a growth-dependent manner and form a layer surrounding the bacteria that enables them to form biofilm and aggregates [11]. PNAG production has been shown to be an essential virulence factor *in vivo* by evading uptake by phagocytic cells such as neutrophils [46]. Hence, the PNAG layer may conceal cell wall components of importance for bacterial endocytosis and/or cytokine induction and may accordingly cause the low cytokine induction observed when stimulating with SP bacteria. It was previously shown that the *S*. *aureus* strain 15981 produces high amounts of PNAG and that this production is abrogated in a mutant strain of 15981 lacking *ica* [11,31]. The SP *S*. *aureus* 15981 produced aggregates, which were not present in the *ica* mutant (Fig 6A), confirming that the PNAG influences the phenotype of the bacteria. Most importantly, in line with previous findings [11], this aggregation was not only present in the SP but also in the EP, albeit to a lesser extent. To assess the role of PNAG in the induction of IL-12 in the DCs, we thus compared the capacity of *S*. *aureus* 15981 WT and the *ica* mutant to elicit IL-12 production. DCs subjected to the strain 15981 mutant lacking *ica* in EP resulted in a much more potent IL-12 production compared to the WT (Fig 6B), demonstrating that already in the EP the produced PNAG may affect the accessibility of structures of importance for IL-12 stimulation. Also, endocytosis of the *ica* mutant was a prerequisite for IL-12 induction (Fig 6C). There was no difference in the SP-induced IL-12 production between the two strains. TNF-α production was significantly increased when DCs were stimulated with the EP *ica* mutant, whereas the SP mutant showed a decreased capability to induce TNF-α compared to the WT strain, signifying that in EP, other molecules than PNAG, was involved in the altered IL-12 and TNF-α inducing capacity. The IL-10 response after stimulation with the EP and SP *ica* mutant strain, respectively, was significantly lower than after stimulation with the WT *S*. *aureus* 15981 (Fig 6B). In summary, already in the EP, PNAG production affects the capability of *S*. *aureus* to induce strong IL-12 responses; however, the presence of PNAG is not alone sufficient to cause the non-stimulatory phenotype of SP *S*. *aureus*.

### IL-12 induction is dependent on D-alanylation of teichoic acids

**Fig 7:**
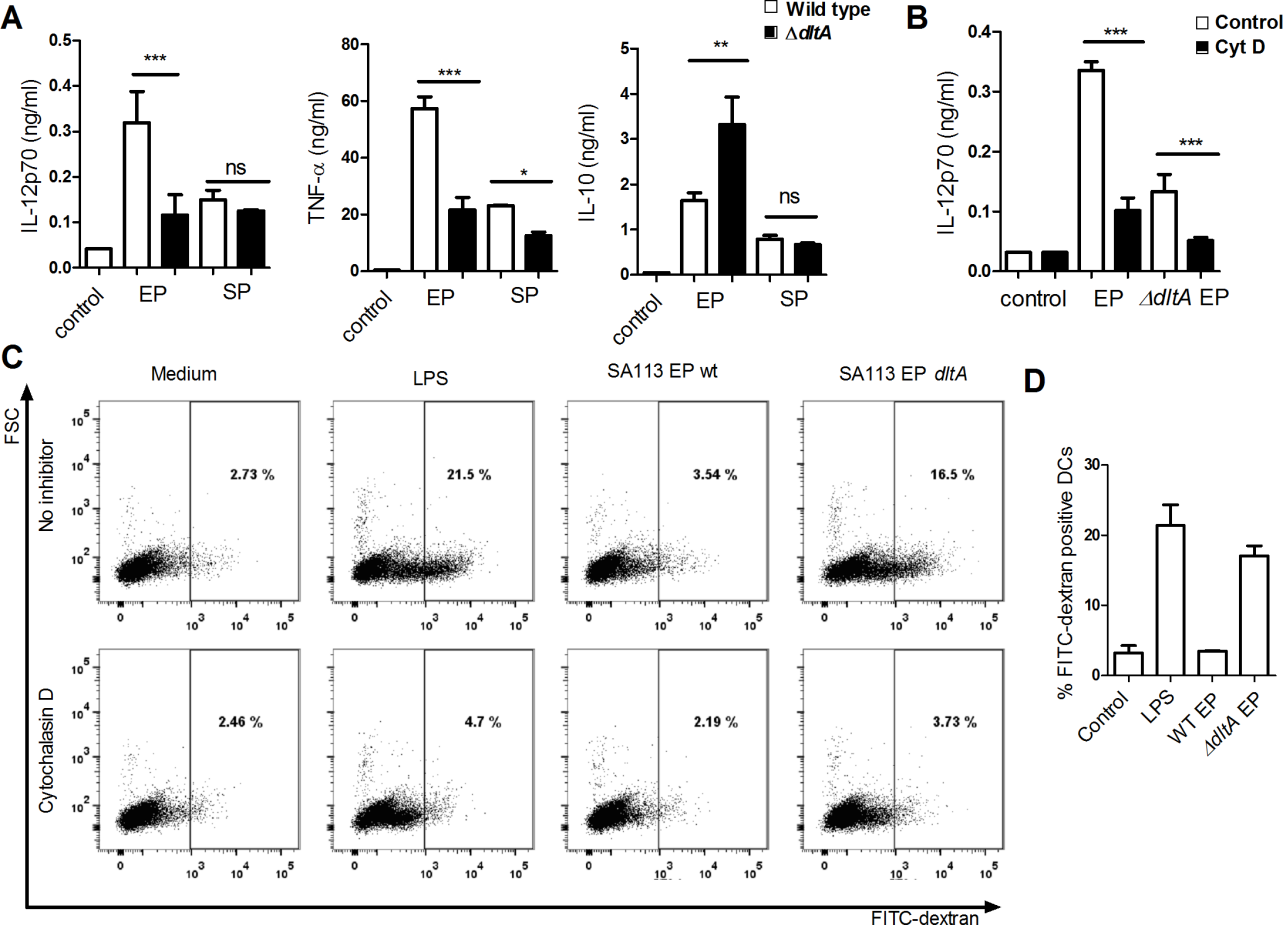
D-alanylation prevents induction of macropinocytosis and is required for IL-12 production. (A) DCs were stimulated with SA113 or its isogenic *dltA* mutant in exponential or stationary phase. IL-12p70, TNF-α and IL-10 were measured in the supernatant after 20h. (B) Cytochalasin D (0.5 μg/ml) treated DCs were stimulated with SA113 and its isogenic *dltA* mutant in exponential phase and the levels of IL-12p70 were measured in the supernatant after 20h. (C) DCs were treated with cytochalasin D for 1h and were subsequently subjected for 30 min to *S*. *aureus* SA113 or its isogenic *dltA* mutant both in EP. Next the cells were chased with FITC-dextran for 10 min, and the uptake of dextran was measured by flow cytometry and plotted against FSC. (D) Combination of three technical replicates of C. Means and SD are based on technical replicates. Data represents one experiment out of three.

Pre-treatment with LTA, in contrast to the tested TLR-ligands; LPS, and PamCSK, and to PGN increased the EP-induced IL-12 production, despite not being able to induce IL-12 *per se*. We therefore speculated whether LTA is involved in endocytosis of *S*. *aureus*, while other molecules, once *S*. *aureus* is present in the endosome compartment, from here promote the IL-12 stimulatory signaling. In wild type *S*. *aureus*, the majority (~70%) of LTA and wall teichoic acid is D-alanylated [27] and the expression of genes involved in D-alanylation are up-regulated during EP [5], hence we speculated that D-alanylated teichoic acids plays a key role in the IL-12 induction, by facilitating phagocytosis of the bacteria and/or inhibiting the TLR2 stimulation. To address the role of teichoic acids and its D-alanylated form, we stimulated DCs with strain SA113 mutated in *dltA*, the D-alanine carrier ligase, rendering it unable to incorporate D-alanine into the teichoic acids (Fig 7). Lack of D-alanylation in the EP resulted in a dramatically lower induction of IL-12 compared to the wild type strain (Fig 7A), demonstrating a key role of D-alanylated teichoic acids for a potent IL-12 induction in DCs. In contrast, the SA113 *dltA* induced higher IL-10 production compared to the wild type strain. No differences were observed between the mutant and wild type strains in SP for IL-12 or IL-10, whereas TNF-α required D-alanylated teichoic acids to some degree for the stimulatory effect in both growth phases. Phagocytosis of both the wt and *dltA* mutant in EP was required for IL-12 induction (Fig 7B). Intriguingly, in contrast to the wild type, the SA113 *dltA* mutant strain significantly increased macropinocytosis to a level comparable of that induced by LPS. (Fig 7C and 7D). Taken together, these data signify the importance of D-alanylation of LTA for inhibiting macropinocytosis and, indirectly, for induction of IL-12.

## Discussion

IL-12 produced by DCs during staphylococcal infection has been shown to be crucial for the activation and regulation of immune cells, and hence for optimal bacterial clearance [14]. The development of IFN-γ producing Th1 cells has been reported to reduce the mortality rate and bacterial burden in mice and is highly dependent on the production of IL-12 [15, 16]. Earlier studies reported that *S*. *aureus* is unable to induce significant IL-12 production in human monocytes and monocyte-derived DCs [47–49] and in immature DCs derived from the mouse spleen [14]. Interestingly, splenic DCs from *S*. *aureus* infected mice were fully capable of producing IL-12 upon ex-vivo stimulation [14], indicating that *S*. *aureus* may be capable of inducing a IL-12 stimulating state under *in vivo* conditions. We therefore aimed to investigate whether growth phase of *S*. *aureus* was important for IL-12 production. Not much attention has been given to the growth phases that *S*. *aureus* naturally will go through during infection and how this may influence the immunological clearance of bacteria. Indeed, we found that IL-12 induction was dependent on growth phase, with EP bacteria being much more potent DC IL-12 inducers than the SP bacteria. Inactivation of the bacteria with UV did not affect this outcome appreciably, indicating that it is not secreted compounds that lead to the IL-12 production. In line with other studies, we observed that the induction of cytokines was dependent on recognition of intact bacteria and not cell wall fractions, as lysostaphin treatment of *S*. *aureus* significantly reduced the IL-12 response and this was further supported by the finding that inhibition of endocytosis abrogated IL-12 production. Phagocytosis and endosomal acidification have previously been shown to be essential for TNF-α production in macrophages upon stimulation with *S*. *aureus* [38]. This is in line with our results, were we show that Cytochalasin D, bafilomycin 1 or chloroquine treated DCs were unable to produce TNF-α and IL-12, while IL-10 was shown to be induced independently of actin-dependent endocytosis, indicating that IL-10 can be induced through stimulation directly from the cell surface as well as through endosomal stimulation. Inhibition of acidification, however, did lead to halt of *S*.*aureus* but not of LPS induced IL-10 production indicating that if endocytosis takes place acidification is required for efficient cytokine induction. In contrast, acidification by bafilomycin 1 but not by chloroquine increased the LPS induced cytokine production. Chloroquine is known to interfere with many steps in signaling pathways including inhibition of ERK activation [50] and eicosanoid production [51], which may explain the different effects of the two inhibitors. In summary, our data indicate that a two-step sequence is required for the induction of IL-12 by the EP *S*. *aureus*; first the bacteria have to be taken up, next the bacteria have to stimulate IL-12 induction from the endosome.

DCs are equipped with PRRs that recognize specific ligands expressed by the pathogen, and drive a specific immune response based on the receptors that are involved [52]. Depending of the type, the PRR is present in the plasma membrane on the surface of DCs and/or in the endosome and facilitate signal transduction from here. TLR ligands such as LPS and Pam3CSK4 have previously been shown to bind to TLRs present on the plasma membrane and accordingly to enhance macropinocytosis, which is characterized by an actin dependent nonspecific non-receptor mediated uptake [39]. Also the Gram negative *E*. *coli* was capable of inducing enhanced macropinocytosis signifying the presence of TLR-ligands (e.g. LPS) on its surface (unpublished data). Neither the EP nor SP *S*. *aureus* induced enhanced macropinocytosis, indicating the absence of available TLR-ligands on the surface of *S*.*aureus*. The bacterial cell wall components, PGN and LTA have previously been reported to stimulate through TLR2 [41–43], although the TLR2 stimulating of LTA and PGN is highly debated and several studies have shown that the TLR stimulating activity is caused by lipoprotein or LPS [25–26, 44–45]. We found that while PGN stimulated enhanced macropinocytosis, the *S*. *aureus* LTA was unable to do so. Concomitantly, pretreatment with PGN but not LTA abrogated the IL-12 induction. This indicates that PGN, or rather the TLR2 binding component of the PGN preparation, is not available in intact *S*.*aureus* for ligation with TLR2 on the plasma membrane, as we showed that in contrast to stimulation with *S*.*aureus*, isolated PGN enhanced macropinocytosis and was able to abrogate IL-12 induction. However, as our data clearly show that uptake of the bacteria, which is also increased by increased macropinocytosis, is mandatory for the IL-12 induction, it cannot be the enhanced macropinocytosis as such that causes the abrogation of the IL-12 response. Rather, the activation of the TLR in the plasma membrane, leading to the recruitment of adaptor proteins for signaling could be involved. TLR2 and TLR4 in the plasma membrane both recruit the adaptor protein MyD88 for signal transduction in collaboration with Mal, when ligated with e.g. PamCSK or LPS [53,54]). In contrast, when ligated within the endosome, TLR4 employs the adaptor protein TRIF, and leads to induction of I*fn*β expression [55]. The adaptor protein(s) involved in endosomal TLR2 signaling remains to be identified; however, we have recently shown that MyD88, but not Mal, was involved in the endosomal activation of *Ifnβ* expression through stimulation with *L*. *acidophilus* NCFM [56]. This indicates that MyD88 may be involved in both plasma membrane and endosomal TLR2 signaling, but that the signaling pathways yet differ due to employment of different co-adaptor molecules. To this end, it is important to remember that IFN-β in turn induces IL-12 production in the DCs [19]. We showed here that *S*. *aureus* was indeed capable of inducing *Ifnβ* expression, hence the IL-12 production may, at least in part, be induced through the action of IFN-β. Most interestingly, stimulating with the *dltA* mutant that is unable to incorporate D-ala into LTA in the *S*. *aureus* cell wall resulted in an enhanced macropinocytosis and concomitant abrogation of IL-12 induction. Hence, we suggest that the dealanylated, but not the alanylated form of LTA, stimulates induction of increased macropinocytosis or, alternatively, exposes other molecules in the cell wall for this stimulation. As the TLR ligands LPS and Pam3CSK4 also stimulate increased macropinocytosis, we speculate that the cell was component might be lipoprotein stimulating through TLR2. Such stimulation could recruit MyD88 to the membrane leading to TLR2-MyD88-Mal activation, which may inhibit later MyD88 recruitment the endosomes. Of note, the alanylated LTA is rather readily hydrolyzed to LTA, e.g. under conditions as those present in the mature endosome [24]. Accordingly, it is likely that endosomal dealanylation takes place in the endosome giving rise to TLR stimulation and induction of *Ifn-β* expression and in turn, IL-12 induction. This is supported by the fact that addition of Chloroquine, and also Bafilomycin A, a specific inhibitor of vacuolar proton ATPases, (data not shown), abrogated the IL-12 production. As mentioned earlier, any TLR2 ligands released by the degradation of the bacteria and other PRRs may be involved. To this end, it should be kept in mind that LTA was not able to stimulate IL-12 production *per se*, thus other molecules are likely to be involved in the IL-12 induction, whereas D-alanylated LTA may play a key role in the phagocytosis of the bacteria.

We also demonstrated that expression of PNAG affected the capability of *S*. *aureus* to induce IL-12, as stimulation with the *ica* deficient strain, which fails to produce PNAG, increased the production of IL-12 compared to the wild type strain, however only when harvested in EP. No difference was observed between the strains during SP. These data may suggest that important PRR ligands present in EP are partially hidden beneath the PNAG layer, thus indicating that only in the very early exponential phase the *S*. *aureus* is capable of inducing a potent IL-12 response of key importance for an efficient clearance *in vivo*. In contrast, the PNAG depletion did not show any effects on the IL-12 induction when stimulating with the SP bacteria. Hence, in the SP other factors than PNAG affect the IL-12 inducing capacity of *S*. *aureus*. It is known that vast changes take place in the expression of genes during the transition from EP to SP in *S*. *aureus*. Other polysaccharides than PNAG are produced that may also conceal the bacterial stimulatory molecules are being produced [9]. In addition the expression of many other molecules present in the EP is down-regulated. Thus, many other factors may through either their presence or absence play roles in the absent IL-12 induction seen in the stimulation with SP bacteria.

In summary, the results reported here show that EP *S*. *aureus* stimulates DCs to induce IL-12 more strongly than SP. This stimulation is dependent on uptake of the bacteria and some endosomal degradation of the bacteria is required for the IL-12 induction. In addition, the absence of TLR-ligands on the surface of EP *S*. *aureus* seems to be mandatory for the endosomal activation to take place, as lack of alanylation gives rise to increased macropinocytosis and loss of IL-12 induction. Also PNAG, known to form a capsule, inhibited the IL-12 induction by SP *S*.*aureus*, probably by concealing stimulatory molecules of importance for the IL-12 production. These data provide new insight into how *S*. *aureus* in the early phase of infection may induce IL-12 production of importance for an efficient bacterial clearance, and also how the bacteria may evade such clearance; a knowledge that may contribute to the development of new therapeutic strategies.

## Supporting Information

S1 Appendix Data showed in Figs 1–7. (RAR)
